# Early Predictors of Disability and Cognition in Multiple Sclerosis Patients: A Long-Term Retrospective Analysis

**DOI:** 10.3390/jcm12020685

**Published:** 2023-01-15

**Authors:** Eleonora Virgilio, Domizia Vecchio, Maria Francesca Sarnelli, Valentina Solara, Roberto Cantello, Cristoforo Comi

**Affiliations:** 1 Neurology Unit, Maggiore Della Carità Hospital, Department of Translational Medicine, University of Piemonte Orientale, 28100 Novara, Italy; 2 Neurology Unit, S. Andrea Hospital, Department of Translational Medicine, University of Piemonte Orientale, 13100 Vercelli, Italy

**Keywords:** multiple sclerosis, cognition, disability predictors, progression, neurodegeneration, biomarkers

## Abstract

We conducted a retrospective analysis on multiple sclerosis (MS) patients with perceived cognitive decline and long disease duration to investigate early predictors of future cognitive impairment (CI) and motor disability. Sixty-five patients complaining of cognitive decline were assessed with an extensive neuropsychological battery at the last clinical follow-up and classified as mildly impaired, severely impaired, and cognitively spared based on the results. Motor disability was assessed with EDSS, MSSS, and ARMSS. Baseline demographic, clinical, and imaging parameters were retrospectively collected and inserted in separate multivariate regression models to investigate the predictive power of future impairment. Twenty-one patients (32.3%) showed no CI, seventeen (26.2%) showed mild CI, and twenty-seven (41.5%) showed severe CI. Older and less educated patients with higher EDSS, longer disease duration, and higher white matter lesion load (WMLL) at diagnosis (particularly with cerebellar involvement) were more likely to develop CI after a mean follow-up from diagnosis of 16.5 ± 6.9 years. DMT exposure was protective. The multivariate regression analyses confirmed WMLL, disease duration, and educational levels as the parameters with significant predictive value for future CI (R2 adjusted: 0.338 *p*: 0.001). Older patients with progressive phenotype both at diagnosis and T1 were more likely to be not fully ambulatory at T1 (R2 adjusted: 0.796 *p*: 0.0001). Our results further expand knowledge on early predictors of cognitive decline and evolution over time.

## 1. Introduction

Multiple sclerosis (MS) disease courses are highly variable, with patients presenting a progressive disease course from onset and patients with relapse onset. Moreover, MS patients experience differences in the rate of disease progression, accumulation of disability, and development of certain invalidating symptoms such as cognitive impairment (CI). Currently, limited information is available at diagnosis to predict MS patients’ long-term evolution. The introduction of disease-modifying therapies (DMTs) has also contributed to observing evolution in the natural history of the disease, reducing relapse rate, but minimally impacting on progression [1]. In the long term, the shift to a secondary progressive (SP) phase is one of the primary determinants of motor disability in relapse-onset MS. Natural history studies defined that 50% of relapsing-remitting (RR) patients [2] or even fewer [3,4] will develop SP MS in the first 15 years after the onset. Identifying patients with a shorter latency to SP onset would help select appropriate DMT strategies. Moreover, the exact pathological mechanisms underlying the transition to the SP phase are not fully understood [5].

In the past, clinical and radiological factors have been recognized to be predictive for long-term outcomes, especially male gender, higher age at disease onset, pyramidal symptoms and incomplete recovery from the first clinical attack, shorter time interval to second clinical attack, number of relapses in early disease phase, higher radiological dissemination in space and early brain atrophy development. However, the disease heterogeneity makes prognostic statements at onset challenging [6,7,8,9].

CI is also an essential source of disability in MS patients, impacting the quality of life, employment, and treatment compliance. CI can occur from early disease stages and worsen over time [10,11,12], but some patients remain cognitively spared even after a long disease duration. Some domains are more likely to be impaired in the MS population (e.g., information processing speed) [11,12,13], but CI can be highly variable, similar to other neurological manifestations. It is influenced by many factors such as genetics, sex, intelligence, and disease course [14] and may differ significantly between progressive and RR disease [14,15]. The natural history of CI remains to be elucidated entirely: patients with a high motor disability can be cognitively spared, whereas patients without a physical disability can develop CI after a long disease course. In the clinical setting, the most common neuropsychological test battery focuses on screening information processing speed, and verbal and visuospatial memory, i.e., in the BICAMS test battery, which is very helpful to screen cognition in early disease stages but can miss some cognitive dysfunction [10]. Moreover, the relationship between demographic, clinical variables, conventional MRI measures available in clinical practice, and long-term cognition is unresolved.

Based on these premises, there is a demand for robust prognostic markers in early disease phases to identify patients with a higher risk for disease progression and development of CI [16]. Those markers may help define a future tailored treatment. We aimed to extensively explore the cognitive status of an MS cohort with a long disease course and identify early predictors of long-term outcomes, including physical disability and CI.

## 2. Materials and Methods

### 2.1. Study Population

We retrospectively selected patients with MS diagnosis according to the McDonald Criteria [17,18], including both bout and primary progressive onset, who underwent an extensive neuropsychological (NP) test battery in our MS Centre from January 2017 to December 2018. NP evaluation was performed because of subjective CI complaints during annual clinical follow-up. A total of 90 MS patients were tested. To assess long-term cognition and disability predictors, we excluded patients with less than 10 years of disease duration and patients with no available MRI and/or complete clinical records detailing characteristics from disease onset. Patients with history of medical or psychiatric disorders that could affect cognitive function, patients older than 65 years at the recruitment, and patients with drug or alcohol abuse, relapse, or corticosteroid treatment within the previous month of the cognitive evaluation were also excluded. The final analysis was performed on a group of 65 MS patients. The demographic, clinical, and radiological information from the onset and diagnosis were retrospectively collected (T0). T1 corresponds to the last clinical follow-up when the NPS evaluation was performed. The study was conducted according to the Declaration of Helsinki. Prospective informed consent, ethical review, and approval were waived due to the study’s retrospective nature and anonymized data. No risks were expected for subjects considering the retrospective study design, and the study results did not impact on the diagnosis or management of study participants. The datasets generated during the analysis of this study are available from the corresponding author upon reasonable request.

### 2.2. Clinical Assessment

At the evaluation time, the disease course was defined according to 2010 or 2017 McDonald criteria and the Lublin definition [17,18,19]. Patients were evaluated using an extensive NP battery, including 11 tests to assess multiple domains such as attention, executive functions, verbal fluency, and information processing speed. Depression was also tested with the Beck Depression Inventory (BDI-II) [20] (Table 1). Raw scores were corrected according to normative values to adjusted and equivalent values [21,22,23,24,25,26], and patients were defined as cognitively impaired if at least two tests found severe alteration and mild CI if only one test discovered severe impairment. Two trained and certified neuropsychologists (MFS and VS) performed the cognitive evaluation. At the NP evaluation, two trained neurologists (EV and DV) calculated the most used disability score for MS patients: the Expanded Disability Status Scale (EDSS) [27]. We also calculated, retrospectively, two further disability scores: the MS Severity Score (MSSS) [28] and the age-related MSSS (ARMSS) [29]. We collected clinical and demographic data on disease onset and diagnosis (sex, age, type, and recovery from first attack, annualized relapse rate of the first five years), presence of cerebrospinal fluid oligoclonal bands, MRI white matter lesion load at diagnosis categorized in low or high, spinal cord involvement, and gadolinium-enhancing lesion.

### 2.3. Statistical Analysis

We produced an anonymized dataset using collected data and performed statistical analysis using SPSS 25.0 for Windows (SPSS Inc., Chicago, IL, USA) and GraphPad Prism 9 for Windows (GraphPad Software, La Jolla, CA, USA). Categorical data are presented with median, range, and interquartile range (IQR), proportions as numbers and percentages, and continuous data with mean and standard deviation (SD). We used Mann-Whitney and Kruskal-Wallis tests to compare continuous variables, the Chi-Squared test with Yates’s correction when appropriate, and the Fisher test for categorical variables. Spearman’s rank correlation coefficient test was used to evaluate the correlation between continuous variables and partial correlation with correction for EDSS and MRI status. Linear regression analyses, including parameters at baseline as independent variables and normalized cognitive scores and disability scores as dependent variables, were run to identify the best predictors of CI and motor disability. All tests were two-sided, and the significance threshold was set to *p* < 0.05.

## 3. Results

### 3.1. Predictors of Cognitive Impairment

The baseline and T1 characteristics of the study cohort are shown in Table 1 and Table 2. We enrolled patients with at least 10 years of follow-up from diagnosis. Mean disease duration from onset was 16.5 ± 6.9 years (median 14 years). Mean disease duration from diagnosis was 12.8 ± 6.8 years (median 11 years).

Eleven out of sixty-five patients (17%) developed an SP disease course during follow-up. Fifty patients (76.9%) remained as RR MS, and four (6.1%) showed a progressive disease course from the diagnosis (PPMS). Almost half of our population at baseline showed high dissemination in space at MRI with spinal and/or cerebellar involvement. EDSS and disability scores significantly increased over time (EDSS at diagnosis vs. EDSS at T1 *p* < 0.0001). The mean disease duration from the onset of our cohort was 16.5 ± 6.9 years. At T1, only twenty-one patients (32.3%) showed no CI, seventeen (26.2%) mild CI, and twenty-seven (41.5%) severe CI. The most common altered tests were PASAT (30.7%), Digit span (39.9%), and Corsi span (36.9%), whereas SDMT was altered only in the 12.3% of the cohort and the less-altered tests were phonetic and semantic fluency.

We compared patients with and without CI and with mild and severe CI. All the results are reported in Table 3.

Patients with CI were older (*p*: 0.04 and *p*: 0.05), less educated (*p*: 0.0008 and 0.001), with longer disease duration from onset and diagnosis (*p*: 0.008, *p*: 0.003 and *p*: 0.01) and with higher BDI scores (*p*: 0.03). No differences were observed between males and females in terms of cognition. The ARR, the type of onset, or phenotype at diagnosis, did not influence cognition over time, whereas patients with at least one cerebellar lesion were more likely to develop CI over time (*p*: 0.02, *p*: 0.03 and odds ratio 0.24–95%CI 0.07–0.76). Similarly, high WMLL also displayed the same results (*p*: 0.01, *p*: 0.02 and odds ratio 0.25–95%CI 0.07–0.76). Patients with cerebellar involvement performed poorly, particularly in tests for information processing speed, verbal and visuospatial short-term memory, and working memory (Table S1). BDI was also higher (*p*: 0.02). On the contrary, patents with or without spinal lesions at baseline performed similarly at NPS evaluation (*p* > 0.05 when compared every single test). Patients with spinal cord lesions performed poorly, particularly for the Corsi test, Weigl test, and short story test, but similarly to patients without spinal lesions. Patients with low brain lesion load showed mean equivalent results above or sometimes at the inferior limit of normality except for PASAT2 (which account for information processing speed). However, it was less impaired than in patients with high WMLL. At T1, thirty-five (53.8%) patients were under DMT (twenty-four (36.9%) with low-efficacy DMT and eleven (16.9%) with high-efficacy DMT); of the other thirty (46.2%) patients, twenty (30.8%) had never been exposed to DMTs, whereas ten (15.4%) patients where previously under immunosuppressive treatment, then stopped for various reasons. We observed a protective effect of the presence of DMT at T1 on cognition (*p*: 0.03, *p*: 0.04 and odds ratio 3.7–95%CI 1.2–10.7) and any exposure of DMT (odds ratio 2.21–95%CI 0.64–6.82; *p*: 0.005). No further analyses were performed due to high heterogeneity in DMT exposure. The majority of patients diagnosed with RRMS at T0 and all the patients with PPMS later developed CI at the NPS evaluation at T1 (*p* > 0.05). The patients who did not transition into an SP phenotype at T1 were less likely to develop CI (*p* > 0.05). We then performed a linear regression analysis including variables that significantly differed in patients with or without CI (cerebellar, WMLL, EDSS and age at diagnosis, educational level, disease duration from onset, and DMT exposure) to determine the best predictors of long-term cognition (Supplementary Materials Table S2). A significant regression equation was found with an adjusted R2 of 0.338 (*p*: 0.001). We confirmed WMLL, disease duration, and educational levels as the best predictors of long-term cognition, whereas DMT exposure, age at diagnosis, cerebellar involvement, and EDSS at diagnosis lost their significance.

### 3.2. Predictors of Motor Disability

Univariate analysis and group comparison were calculated between clinical and radiological data and our disability scores at T1 (EDSS, MSSS, and ARMSS). All results are reported in Supplementary Materials (Table S3). A positive correlation was observed between patient age (at onset, diagnosis, and T1) and MSSS (*p*: 0.03, *p*: 0.01 and *p*: 0.005), and age at T1 and EDSS (*p*: 0.03, *p*: 0.01, and *p*: 0.005). EDSS at T1 correlated with disease duration at onset and diagnosis (*p*: 0.01 and *p*: 0.03). RRMS patients without recovery from the first attack developed at T1 higher MSSS and ARMSS (*p*: 0.049 and *p*: 0.03). Progressive MS at diagnosis and after SP conversion showed higher disability with all scores (*p* < 0.0001). Patients with cerebellar involvement and WMLL at T0 are more likely to reach higher disability evaluated with different scores at T1 (*p*: 0.049, *p*: 0.005, and *p*: 0.04). BDI was higher in patients with more disability (considered with all three scores, *p*: 0.003, *p*: 0.049, and *p*: 0.003). Patients exposed to DMT at any time and/or with DMT at T1 showed a trend of lower disability, and EDSS was statistically lower in patients with DMT at T1 (*p*: 0.049).

We then further categorized patients based on ambulatory autonomy, identifying patients as fully ambulatory (corresponding to an EDSS < 4) and not fully ambulatory at T1 (with an EDSS ≥ 4). Forty-nine patients (75.4%) showed EDSS <4 and sixteen (24.6%) patients showed EDSS ≥ 4. Patients with EDSS ≥ 4 showed a higher age at diagnosis and at T1 (*p*: 0.03 and *p*: 0.0008), whereas they showed a similar age at onset. Similarly, patients not fully ambulatory at T1 displayed a longer disease duration from the onset (*p*: 0.03) and were more likely to be a progressive phenotype from the onset and at T1 (*p*: 0.0003 and *p* < 0.00001). When considering radiological characteristics at baseline, we observed that most patients without cerebellar involvement at T1 were fully ambulatory (*p*: 0.006). More patients with EDSS < 4 were treated with DMT at T1 (*p*: 0.003), and had lower BDI results (*p*: 0.03).

A regression analysis with WMLL, cerebellar involvement, age at diagnosis and at T1, EDSS at diagnosis, MS phenotype both at diagnosis and at T1, disease duration from the onset, and DMTs exposure at T1 was run to determine the best predictor of higher EDSS at long-term follow-up. In a highly significant model (R2 adjusted 0.796, *p*: 0.0001), progressive patients, both PP at diagnosis and progressive over time (SP and PP) with a higher EDSS at baseline, were at higher risk of accumulating disability over time (Supplementary Materials Table S4). Similar regression analyses with MSSS (R2 adjusted, 0.792 *p*: 0.0001) and ARMSS (R2 adjusted 0.741, *p*: 0.0001) as the dependent variable were calculated, confirming the results.

## 4. Discussion

Our retrospective study evaluated early predictors of cognitive and motor disability, highlighting and confirming some interesting findings. Mental status is part of the EDSS scale and is often neglected based on the clinical neurological evaluation or perceived impairment reported by the patient, leading to an unprecise quantification over time. Thus, in the MS population, annual screening with the symbol digit modalities test (SDMT) or similarly validated test (i.e., PASAT) is recommended [13], but information processing speed is only one of the possible altered domains [10]. Enrollment in our study was based on complaints of cognitive difficulties. However, only 67.7% showed some decline at NPS evaluation, further supporting the need for a multidisciplinary assessment for anyone complaining of CI to confirm or exclude CI in patient-reported outcomes [30]. Moreover, we confirmed that SDMT or PASAT alone cannot fully grasp the cognitive status of an MS patient and should be integrated with more comprehensive testing when altered, including verbal and visuospatial.

The cerebellum’s role in the disease has been known since Charcot’s first description of the MS triad. Although abnormalities have been known in MS for a long time, more evidence has been collected on cerebellum function and dysfunction in MS patients only in the past two decades. The cerebellum is a preferred site for focal demyelination in the white and grey matter. The extent of tissue damage in terms of atrophy, focal lesions, and connectivity seems to be related to MS severity and CI, especially in progressive patients and with long disease duration [31,32,33,34,35,36,37]. Our study further confirmed the involvement of cerebellar inflammatory dissemination in early phases with both cognitive and motor functions, even though exclusively in the univariate analysis. In our opinion, this merit is further confirmation in the future, particularly to elucidate if MS patients with extensive cerebellar involvement might be prone to develop specific cognitive patterns.

Disease duration, progressive phenotypes, older age, and lower education (possibly associated with lower cognitive reserve) are other well-known predictive factors of motor and cognitive decline over time [14]. Our findings further reinforce those statements. As expected, DMT exposure is protective; however, since the effect of DMTs on cognition is not solid [37], the protective effect of DMT exposure on CI in our cohort is particularly interesting. Of course, future studies are needed to confirm if certain DMTs have an effect in preventing CI. These strategies could be useful not only in more at-risk patients but also in patients with a higher cognitive reserve.

Furthermore, we also reinforce the evidence that patients with CI and motor disability suffer from a lower quality of life, often associated with depressive symptoms [38,39].

Finally, we acknowledge several limitations in our study, particularly linked to the retrospective nature of the analysis, the absence of a cognitive evaluation at diagnosis, and the enrollment of patients with perceived CI (which represents a possible selection bias). Intermediate time points to evaluate early cognitive and motor disability would be useful to investigate and to intercept a long-term loss of function. The study was also constrained by a small sample size, which could have influenced final results. Our finding needs to be replicated in a prospective cohort from diagnosis, including patients with subjective and not subjective CI. Moreover, our study lacks an advanced MRI analysis of the whole brain, of WM, and grey matter volume quantification. Even though Patti et al. reappraised the importance of WMLL at onset as a predictive factor of CI after a 9-year follow-up [40], when combined, focal lesions and atrophy obtain the best predictive value for long-term outcomes. Those analyses need to be integrated in a prospective future study since cognition in MS and transition to the SP phase have been linked to grey matter pathology [41,42]. Finally, further extensive analysis in regard to DMT exposure were not possible due to high heterogeneity of the type, time, and sequence of exposure.

## 5. Conclusions

In conclusion, our data confirm the importance of investigating cognition with extensive NP evaluation particularly in old patients with a long disease duration. DMT exposure is protective not only on motor disability but also on cognition and we support the role of WMLL, particularly infratentorial inflammatory lesions, as well as clinical and demographic characteristics, as early predictors of cognitive and motor long-term disability in the MS population.

## Figures and Tables

**Table 1:** The study population’s demographic, clinical, CSF, and MRI characteristics (N: 65).

Demographic Characteristics	
Age at onset (yrs); mean ± SD	32.02 ± 9.20
Age at diagnosis (yrs); mean ± SD	35.63 ± 9.90
Age at T1 (yrs); mean ± SD	47.9 ± 9.2
Male/Female; n/n, (%)	17/48, (26.1/73.9%)
EDSS at diagnosis; mean ± SD (median; range)	1.15 ± 0.9 (1; 0–4)
EDSS at T1; mean ± SD (median; range)	2.9 ± 2.0 (2; 0–7.5)
Disease duration from onset (yrs); mean ± SD	16.5 ± 6.9
Disease duration from diagnosis (yrs); mean ± SD	12.8 ± 6.1
Educational level (yrs) mean ± SD	12.1 ± 3.5
ARR in the first 5 y	0.5 ± 0.46
MSSS at T1	2.66 ± 2.31
ARMSS at T1	3.82 ± 2.49
MS type at diagnosis (n %)	
RR	61 (93.9%)
PP	4 (6.1%)
MS type at T1 (n %)	
RR	50 (76.9%)
PP	4 (6.1%)
SP	11 (17.0%)
Clinical characteristics at onset (n %)	
Sensory/pyramidal syndrome	34 (52.2%)
Brainstem/cerebellar syndrome	8 (12.3%)
Optic neuritis	15 (23.1%)
Myelitis	8 (12.3%)
MRI characteristics at diagnosis (n %)	
>9 T2 brain lesions	33 (50.7%)
≤v9 T2 brain lesions	32 (49.3%)
Gd+ lesions	32 (49.3%)
Spinal lesions	39 (60.0%)
Cerebellar lesions	26 (40.0%)
IEF	
Type I	4 (6.2%)
Type II	57 (87.7%)
Type III	2 (3.05%)
Type IV	2 (3.05%)
Cognition at T1	
Mild CI	17 (26.2%)
Severe CI	27 (41.5%)
Not impaired	21 (32.3%)

Abbreviations: ARR: annualized relapse rate, EDSS: Expanded Disability Status Scale, IEF: isoelectrofocusing, T1:CI: cognitive impairment, Gd: gadolinium, MS: multiple sclerosis, RR: relapsing–remitting, PP: primary progressive, SP: secondary progressive.

**Table 2:** Neuropsychological evaluation at T1.

Test	Raw (Mean ± SD)	Adjusted (Mean ± SD)	Equivalent (Mean ± SD)	Cut-Off n.v	N/65 (%) Severly Below n.v	N/65 (%) Mildly Below n.v
BDI	11.1 ± 8.8			<13	3/65 (4.6%)	16/65 (24.6%)
Corsi span	5.0 ± 1.3	4.7 ± 1.3	2.7 ± 1.7	≥3.5	10/65 (15.4%)	14/65(21.5%)
Short story	15.8 ± 5.3	14.6 ± 4.8	3.1 ± 1.4	≥7.5	6/65 (9.2%)	8/65 (12.3%)
Digit span forward	5.9 ± 1.2	5.6 ± 1.1	3.3 ± 1.1	≥3.75	2/65 (3%)	6/65 (9.2%)
Digit span backward	4.0 ± 1.1	-	-	≤2	17/65 (26.1%)	9/65 (13.8%)
Phonetic fluency	34.3 ± 10.3	32.2 ± 9.8	2.9 ± 1.2	≥17.35	4/65 (6.1%)	4/65 (6.1%)
Semantic fluency	46.0 ± 12.3	44.2 ± 11.4	3.4 ± 1.0	≥25	2/65 (3%)	4/65 (6.1%)
TMTa	38.7 ± 25.6	35.0 ± 24.8	3.5 ± 1.1	≤93	2/65 (3%)	4/65 (6.1%)
TMTb	90.4 ± 61.4	83.6 ± 63.4	3.3 ± 1.3	≤282	9/65 (13.8%)	4/65 (6.1%)
TMT b-a	54.0 ± 49.7	50.4 ± 49.6	3.3 ± 1.3	≤186	11/65 (16.9%)	4/65 (6.1%)
Cognitive estimates	14.6 ± 4.3	14.5 ± 3.9		≤19	8/65 (12.3%)	3/65 (4.6%)
SDMT	46.5 ± 15.0	45.3 ±12.1	-	≥34.2	8/65 (12.3%)	-
Weigl test	14.0 ± 17.5	11.7 ± 2.9	2.7 ± 1.3	≥4.50	4/65 (6.1%)	8/65 (12.3%)
Raven’s matrices	29.7 ± 5.3	29.2 ± 4.8	3.0 ± 1.1	≥18	1/65 (1.5%)	7/65 (10.7%)
PASAT 2′ (errors)	20.3 ± 14.5		-	≥10	19/65 (29.2%)	1/65 (1.5%)

Abbreviations: BDI: beck depression inventory, SDMT: symbol digit modalities test; TMT: trail making test; PASAT. SD: standard deviation; n.v: normal values.

**Table 3:** Cognition and prognostic factors.

	(a) No CI (N: 21)	(b) CI (N: 44)	*p*-Value (a) vs. (b)	(c) Mild CI (N: 17)	(d) Severe CI (N: 27)	*p*-Value (a) vs. (c) vs. (d)
Age at onset; mean ± SD	31.29 ± 8.60	32.36 ± 31.29	0.5	31.12 ± 8.6	33.15 ± 9.34	0.5
Age at diagnosis; mean ± SD	34.95 ± 10.35	35.95 ± 9.78	0.6	34.94 ± 9.97	36.59 ± 9.79	0.7
Age at T1; mean ± SD	44.6 ± 9.48	49.9 ± 8.3	0.04	48.65 ± 7.22	50.74 ± 9.0	0.05
Sex (% of grand total)						
Female	20.0%	53.85%		34.21%	36.84%	
Male	12.31%	13.85%	0.1	21.05%	7.89%	0.2
Educational level; mean ± SD	14.11 ± 3.71	11.11 ± 3.37	0.0008	12.12 ± 3.10	10.48 ± 3.43	0.001
BDI; mean ± SD	7.85 ± 5.37	12.81 ± 9.73	0.03	13.93 ± 13.05	12.19 ± 7.53	0.1
Dd from onset mean ± SD	13.7 ± 6.67	17.7 ± 6.73	0.008	17.82 ± 7.31	17.67 ± 6.49	0.03
Dd from diagnosis mean ± SD	10.05 ± 3.29	14.11 ± 6.71	0.01	14.00 ± 7.04	14.19 ± 6.63	0.06
ARR mean ± SD	0.51 ± 0.43	0.48 ± 0.49	0.7	0.54 ± 0.65	0.45 ± 0.36	0.9
EDSS at T0; mean ± SD	1 ± 0.8	1.2 ± 1	0.5	1.03 ± 0.99	1.33 ± 1.04	0.6
EDSS at T1; mean ± SD	2.07 ± 1.46	3.27 ± 2.1	0.01	2.94 ± 1.88	3.48 ± 2.31	0.04
MSSS at T1; mean ± SD	2.02 ± 1.83	2.97 ± 2.47	0.2	2.51 ± 2.04	3.25 ± 2.70	0.4
ARMSS at T1; mean ± SD	3.17 ± 1.97	4.14 ± 2.67	0.1	3.92 ±2.51	4.28 ± 2.80	0.3
Recovery from onset (% of grand total)						
Yes	18.46%	43.08%		16.92%	9.23%	
no	13.85%	24.62%	0.7	26.15%	15.38%	0.8
Type of onset (% of grand total)						
Sensory/pyramidal	15.38%	36.93%		10.77%	26.1%	
Brainstem/cerebellar	6.15%	6.15%		1.54%	4.6%	
Optic neuritis	6.15%	16.92%		9.23%	7.7%	
Myelitis	4.61%	7.69%	0.6	4.61%	3.1%	0.5
Type of MS at T0 (% of grand total)						
RR	33.33%	60.32%		55.26%	42.11%	
PP	0%	6.35%	0.2	0%	2.63%	0.4
Type of MS at T1 (% of grand total)						
RR	30.16%	47.62%		20.31%	26.56%	
SP	3.17%	12.70%		4.69%	9.37%	
PP	0%	6.35%	0.1	1.56%	4.69%	0.3
Spinal (% of grand total)						
Yes	23.08%	36.92%		12.31%	24.62%	
No	9.23%	30.77%	0.2	13.85%	16.92%	0.3
Cerebellar (% of grand total)						
Yes	6.15%	33.85%		10.77%	23.08%	
No	25.15%	33.85%	0.02	15.38%	18.46%	0.03
Gd+ (% of grand total)						
Yes	16.92%	32.31%		15.38%	16.93%	
no	15.38%	35.38%	0.7	10.77%	24.61%	0.4
WMLL (% of grand total)						
Yes	9.23%	41.54%		18.46%	23.08%	
No	23.08%	26.15%	0.01	7.69%	18.46%	0.02
DMT at T1 (% of grand total)						
Yes	23.08%	30.77%		13.85%	16.92%	
No	7.69%	38.46%	0.03	12.31%	26.15%	0.04
Any DMT exposure in the past (% of grand total)						
Yes	24.6%	44.62%		20%	16.92%	
No	6.15%	24.62%	0.2	6.15%	26.15%	0.005
OCB (% of grand total)						
Yes	29.23%	58.46%		23.43%	24.37%	
No	3.08%	9.23%	0.9	1.56%	7.81%	0.4

Group comparison; test used: Mann-Whitney, and Kruskal-Wallis tests for continuous variables; Chi-Squared test and Fisher test for categorical variables. Abbreviations: BDI: SDMT: symbol digit modalities test; TMT: trail making test; PASAT. SD: standard deviation; n.v: normal values. T0 = diagnosis; T1 = time of NPS evaluation.

## Data Availability

Data are available upon reasonable request.

## Supplementary Materials

The following supporting information can be downloaded at: https://www.mdpi.com/article/10.3390/jcm12020685/s1, Table S1: Comparison of cognitive performances based on cerebellar involvement at baseline, Table S2: Predictive factors at MS diagnosis for long term cognition. N:65; Table S3: Motor disability and prognostic factors. Group comparison and correlation analyses.; Table S4: Predictive factors at MS diagnosis for long-term motor disability. N: 65.
