# Supplementary material for: Early Predictors of Disability and Cognition in Multiple Sclerosis Patients: A Long-Term Retrospective Analysis
Source: J Clin Med. 2023 Jan 15;12(2):685. doi: 10.3390/jcm12020685 (PMC9864935; doi:10.3390/jcm12020685)
Supplement: Supplementary file 1 [file jcm-12-00685-s001.zip › jcm-2054704-supplementary.pdf]

Supplementary Materials

Table S1. Comparison of cognitive performances based on cerebellar involvement at baseline.

	Patients without cerebellar involvement			Patients with cerebellar involvement			P values*
**Test**	**Raw (mean ± SD)**	**Adjusted (mean ± SD)**	**Equivalent (mean ± SD)**	**Raw (mean ± SD)**	**Adjusted (mean ± SD)**	**Equivalent (mean ± SD)**	
**BDI***	9.2 ±7.8	-	-	14.2±9.5	-	-	0.03
**Corsi Span**	5.2±1.4	4.9 ±1.3	2.9 ± 1.6	4.7±1.0	4.4±1.0	2.4±1.7	0.1
**Short story**	17.4±4.7	16.1 ±4.4	3.4 ±1.2	13.5±5.3	12.5±4.6	2.6±1.6	0.01
**Digit span forward**	6.1±1.1	5.8 ±1.1	3.5 ±0.9	5.5±1.2	5.2±1.0	2.9±1.3	0.08
**Digit span backword**	4.3±1.0	-	-	3.6±1.1	-	-	0.02
**Phonetic fluency**	37.1±10.1	34.4±9.4	3.2±1.1	30.3±9.2	28.8±9.5	2.5±1.3	0.03
**Semantic fluency**	48.2 ±11.6	46.0 ±11.2	3.6±0.9	42.6±12.7	41.4±11.3	3.2±1.1	0.05
**TMTa**	31.7±19.0	27.5±19.2	3.7±0.9	49.0±30.7	46.1±28.1	3.2±1.2	0.02
**TMTb**	79.6±55.5	69.8±59.0	3.6±1.2	109.8±68.0	109.2±64.4	3.0±1.4	0.05
**TMT b-a**	48.1±45.6	42.3±47.3	3.4±1.3	64.9±55.8	64.9±51.6	3.1±1.4	0.2
**Cognitive estimates**	13.5±4.2	13.5±3.6		16.6±3.8	16.24±3.8	-	0.01
**SDMT**	49.4±14.7	48.1±10.7	-	38.4±13.5	37.4±13.2	-	0.02
**Weigl test**	16.0±22.3	12.13 ±2.9	3.1 ±1.1	11.0±2.9	11.05±2.7	2.2±1.2	0.01
**Raven'smatrices**	30.5±5.2	29.9±4.6	3.2±1.1	28.6±5.3	28.3±4.9	2.7±1.1	0.08
**PASAT 2' (errors)**	49.4±14.7	-	-	34.8±8.8	-	-	0.0005

*pvalue obtained with Mann-Whitney test and reffered to differences between equivalent values or when not avaiable between adjusted or raw values

Abbreviations: BDI: SDMT: symbol digit modalities test; TMT: trail making test; PASAT. SD: standard deviation.

Table S2. Predictive factors at Ms diagnosis for long term cognition. N:65

		Beta	95% CI	P value
**Model***	EDSS	-0.048	-0.134 – 0.087	0.469
	Cerebellum	0.135	-0.109 – 0.368	0.282
	WMLL	**0.265**	0.017 – 0.479	**0.036**
	Age	0.052	-0.009 – 0.014	0.667
	D.D	**0.293**	0.005 – 0.035	**0.01**
	E.L	**-0.320**	-0.073 - -0.012	**0.007**
	Any DMTs	-0.102	-0.337 - 0.131	0.382

*Model considering cognition as a dependent variable and EDSS, cerebellum involvement, EDSS, WMLL, and educational level as independent variables. CI was defined with the presence of at least 2 severely altered tests at NPS evaluation at T1. **Abbreviations:** CI: cognitive impairment, DD: disease duration from the onset, DMT: disease modifying treatment, EDSS: expanded disability status score, EL: educational level, Ms: Multiple sclerosis, NPS: neuropsychological, WMLL= white matter lesion load

Table S3. Motor disability and prognostic factors. Group comparison and correlation analyses.

	**a) EDSS<4 (N49)**	**b) EDSS≥4 (N16)**	**p-value a) vs b)**	**MSSS T1 (N65)**	**ARMSS T1 (N65)**	**EDSS T1 (N65)**
**Age at onset;** mean±SD	31.06 ± 9.53	34.94 ± 7.48	0.1	***r:0.26 p:0.03***	r:-0.18 p:0.1	r:0.14 p:0.2
**Age at diagnosis;** mean±SD	34.20 ±10.23	40.0 ± 7.46	***0.03***	***r:0.29 p:0.01***	r:-0.12 p:0.3	r:0.22 p:0.08
**Age at T1;** mean±SD	45.84 ± 9.25	54.19± 5.02	***0.0008***	***r:0.34 p:0.005***	r:0.008 p:0.9	***r:0.38 p:0.01***
**Sex (% of grand total)**				2.7±2.3	3.9±2.5	2.9±2
Female	56.06%	18.18%	0.9	vs 2.4±2.1	vs 3.6±2.4	vs 2.7±1.9
Male	19.70%	6.06%		p0.6	p0.7	p0.6
**Educational level;** mean±SD	12.22±2.82	11.69±3.86	0.7	r:-0.07 p:0.5	r:-0.003 p:0.9	r:0.09 p:0.4
**BDI;** mean±SD	10.09±8.94	14.31±7.28	***0.03***	***r:0.36 p:0.003***	***r:0.24 p:0.049***	***r:0.36 p:0.003***
**Dd from onset** mean±SD	15.41 ± 6.61	19.56 ± 7.15	***0.03***	r:0.02 p0.8	r:0.17 p0.1	***r:0.31 p:0.01***
**Dd from diagnosis** mean±SD	12.24 ± 6.36	14.50 ± 5.06	0.1	r:-0.02 p0.8	r:0.18 p0.1	***r0.26 p0.03***
**ARR mean±SD**	0.47 ± 0.49	0.6 ± 0.32	0.08	r:0.20 p0.1	***p:0.43 p0.006***	r:0.21 p0.09
**EDSS at T0;** mean±SD	0.93± 0.84	1.81± 0.99	***0.003***	***r:0.54 p<0.0001***	***r:0.49 p<0.0001***	***r:0.51 p<0.0001***
**EDSS at T1;** mean±SD	1.85± 0.86	6.03 ±1.04	***<0.0001***	-	-	-
**MSSS at T1;** mean±SD	1.52 ± 1.09	6.13 ± 1.42	***<0.0001***	-	-	-
**ARMSS at T1;** mean±SD	2.74 ± 1.68	7.16 ± 1.30	***<0.0001***	-	-	-
**Recovery from onset (% of grand total)**				2.1±1.7	3.2±2.1	3.4±2.4
Yes	50.7%	10.7%		vs 3.5±2.7	vs 4.7±2.7	vs 2.5±1.6
no	24.6%	13.8%	0.9	***p0.049***	***p0.03***	p0.1
**Type of onset (% of grand total)**						
Sensory/pyramidal	12.3%	40%				
Brainstem/cerebellar	4.6%	7.7%				
Optic neuritis	4.6%	18.5%				
Myelitis	3.1%	9,2%	p:0.8	p:0.7	p:0.2	p:0.6
**Type of MS at T0**						
**(% of grand total)**	75%			2.6±1.9	3.5±2.2	2.6±1.5
RR	18.7%	0%		vs 7.7±0.	vs 8.6±0.2	vs 7.1±0.2
PP		6.2%	***p:0.0003***	***p<0.0001***	***p<0.0001***	***p<0.0001***
**Type of MS at T1**						
**(% of grand total)**	71.8%					
RR	0%	3.1%				
PP	3.1%	6.2%				

SP		15.6%	***p:<0.0001***	***p<0.0001***	***p<0.0001***	***p<0.0001***
**Spinal (% of grand total)**				3.1±2.5	4.1±2.4	3.0±2.0
Yes	43.1%	16.9%		vs 2±1.8	vs 3.2±2.4	vs 2.5±1.8
No	32.3%	7.7%	p:0.4	p0.06	p0.09	p0.2
**Cerebellar (% of grand total)**				3.5±2.7	4.8±2.5	3.6 ±2.3
Yes	23.1%	16.%		vs 2.0±1.8	vs 3.1±2.2	vs 2.3 ± 1.6
No	52.3%	7.7%	***p:0.006***	***p0.049***	***p0.005***	***p0.040***
**Gd+ (% of grand total)**				2.6±2.3	3.4±2.4	2.8±1.9
Yes	33.8%	15.4%		vs 2.7±2.3	vs 4.2±2.4	vs 2.9±2
no	41.5%	9.2%	p:0.2	p0.7	p0.2	p0.8
**WMLL (% of grand total)**				3.2±2.5	4.3±2.5	3.2±2.1
Yes	36.9%	13.8%		vs 2-0±1.9	vs 3.2±2.4	vs 2.5±1.8
No	38.5%	10.8%	p:0.6	***p0.03***	***p0.049***	p0.08
**DMT at T1 (% of grand total)**				2.1±1.7	3.4±2	2.3±1.5
Yes	46.9%	7.6%		vs 3.3±2.7	vs 4.2±2.8	vs 3.4±2.4
No	28.7%	16.7%	***p:0.03***	p0.07	p0.3	***p0.049***
**Any DMT exposure in the past (% of grand total)**						
Yes				2.5±2 vs	3±2.3	2.5±1.9
No	51.5%	18.2%		2.8±2.4	vs 4.1±2.4	vs 3±2
	24.2%	6.1%	p:0.5	p0.4	p0.08	p0.4
**OCB (% of grand total)**						
Yes	65.6%	21.9%		3±2vs2.5±1.5	2.7±2.4 vs 2.1±1.6	3.9±2.5vs 3.2± 2.3
No	10.9%	1.6%	p:0.4	p0.7	p0.8	p0.5

Group comparison; test used: Mann–Whitney and Kruskal–Wallis tests for continuous variables; Chi-Squared test and Fisher test for categorical variables. **Abbreviations:** ARMSS: age-related MSSS, ARR: annualized relapse rate, DD: disease duration, DMT: disease-modifying treatment, EDSS: expanded disability status score, Gd: gadolinium, OCB: oligoclonal bands, Ms: Multiple sclerosis, MSSS: MS severity score, WMLL: white matter lesion load

Table S4. Predictive factors at Ms diagnosis for long-term motor disabilty. N:65

		Beta	95% CI	P value
**Model***	MS Type at T0	0.500	1.890-4.709	0.000
	MS Type at T1	0.192	-0.833 – 1.371	0.042
	DMT at T1	0.038	-0.212 – 0.420	0.513
	DD from onset	-0.001	-0.041 – 0.040	0.987
	Cerebellum	0.075	-0.187 – 0.798	0.219
	WMLL	0.031	-0.343 – 0.592	0.597
	Age at diagnosis	-0.084	-0.061 - 0.027	0.438
	Age at T1	0.145	-0.018 – 0.083	0.204
	EDSS at T0	0.239	0.251 – 0.761	0.000

*Model considering EDSS at T1 as a dependent variable and EDSS at diagnosis, cerebellum involvement, WMLL, MS phenotype at diagnosis and T1 , DMT at T1, disease duration from the onset, age at diagnosis, and at T1 as multiple independent variables. **Abbreviations:** DD: disease duration, DMT: disease-modifying treatment, EDSS: expanded disability status score, Ms: Multiple sclerosis, WMLL= white matter lesion load
